# Dextromethorphan Enhances Apoptosis and Suppresses EMT in PANC-1 Pancreatic Cancer Cells: Synergistic Effects with Gemcitabine

**DOI:** 10.3390/ijms26178151

**Published:** 2025-08-22

**Authors:** Gulsah Medet, Ahmet Inal

**Affiliations:** 1 Department of Pharmacology, Institute of Health Sciences, Erciyes University, Kayseri 38039, Turkey; eczgulsahmedet@gmail.com; 2 Department of Pharmacology, School of Medicine, Erciyes University, Kayseri 38039, Turkey

**Keywords:** dextromethorphan, NMDA receptor antagonist, PANC-1, pancreatic cancer, apoptosis, EMT

## Abstract

This study aimed to evaluate the effects of dextromethorphan (DX), alone and in combination with gemcitabine (GEM), on cell viability, apoptosis, and epithelial–mesenchymal transition (EMT) markers in PANC-1 human pancreatic cancer cells. PANC-1 human pancreatic cancer cells were cultured and treated with varying concentrations of dextromethorphan (DX), gemcitabine (GEM), and 5-fluorouracil (5-FU), both as monotherapies and in combination. Cytotoxic effects were assessed using the MTT assay, and IC_50_ values were calculated at 24, 48, and 72 h. Apoptotic responses were evaluated using Annexin V-FITC/PI staining followed by flow cytometry. Protein expression levels of Bax, Bcl-2, and Vimentin were determined via immunocytochemistry, while EMT markers (E-cadherin, N-cadherin, Vimentin) were analyzed using flow cytometry. Relative mRNA expression of apoptotic and EMT-related genes was quantified by qRT-PCR. DX exhibited time- and dose-dependent cytotoxicity in PANC-1 cells, with IC_50_ values of 280.4 µM at 24 h, 163.2 µM at 48 h, and 105.6 µM at 72 h. For GEM, the 72 h IC_50_ was 57.53 µM. The combination of DX 50 µM + GEM 12.5 µM resulted in significantly lower cell viability (24.93 ± 3.12%) compared to GEM 25 µM (35.33 ± 5.22%) and DX 100 µM (51.40 ± 3.10%) (*p* < 0.001). Flow cytometry revealed significant increases in early (21.83 ± 1.32%) and late apoptotic cells (32.20 ± 0.84%) in the combination group, with a corresponding reduction in viable cells compared to control (24.93 ± 3.12% vs. 89.53 ± 0.97%, *p* < 0.001). Immunocytochemical analysis showed increased Bax-positive cell count (62.0 cells/unit area), and decreased Bcl-2 (19.0) and Vimentin (28.0) levels in the combination group compared to control (Bax: 15.0, Bcl-2: 60.0, Vimentin: 70.0) (*p* < 0.001). Flow cytometry for EMT markers demonstrated increased E-cadherin (83.84 ± 0.65%) and decreased Vimentin (71.04 ± 1.17%) and N-cadherin (30.47 ± 0.72%) expression in the DX + GEM group compared to EMT control (E-cadherin: 68.97 ± 1.43%, Vimentin: 91.00 ± 0.75%, N-cadherin: 62.47 ± 1.13%) (*p* < 0.001). qRT-PCR supported these findings with increased Bax (2.1-fold), E-cadherin (2.0-fold), and reduced Bcl-2 (0.3-fold) and XIAP (0.6-fold) in the combination group (*p* < 0.05). Dextromethorphan, particularly in combination with gemcitabine, appears to enhance apoptosis and suppress EMT-associated marker expression in PANC-1 cells, supporting its potential as an adjuvant agent in pancreatic cancer therapy.

## 1. Introduction

Pancreatic cancer is one of the most lethal malignancies, ranking as the seventh leading cause of cancer-related deaths globally. The 5-year survival rate remains below 10% due to its aggressive nature, late diagnosis, and limited therapeutic response [1]. Although surgical resection is the primary curative approach, most patients present with inoperable disease, making systemic chemotherapy an essential part of the treatment strategy [2].

Gemcitabine (GEM) and 5-fluorouracil (5-FU) are among the most commonly used chemotherapeutic agents in pancreatic cancer, yet resistance and limited efficacy remain significant obstacles [3]. Therefore, there is an urgent need for novel adjuvant therapies that can improve the response to existing treatments and target key cellular mechanisms involved in cancer progression.

Apoptosis resistance and epithelial–mesenchymal transition (EMT) are two fundamental processes implicated in tumor survival, invasion, and metastasis in pancreatic cancer [4]. Apoptosis, or programmed cell death, is often dysregulated in malignancies, leading to unchecked proliferation and therapy resistance [5]. EMT, characterized by the loss of epithelial markers such as E-cadherin and the acquisition of mesenchymal markers like N-cadherin and Vimentin, contributes to increased invasiveness and metastatic potential [6].

N-methyl-D-aspartate (NMDA) receptors, traditionally studied in the central nervous system, have recently been identified in various peripheral tissues and tumor types, including pancreatic cancer [7]. These receptors, particularly the GluN1 and GluN2 subunits, have been shown to play roles in cellular proliferation, migration, and apoptosis [8]. Dextromethorphan (DX), a widely used antitussive agent, is a non-competitive NMDA receptor antagonist with a well-established safety profile. Emerging evidence indicates that NMDA receptor antagonists may exert antiproliferative effects in various cancer cell lines [9,10,11].

A study by North et al. (2017) confirmed the presence of GluN1 and GluN2B subunits in the PANC-1 pancreatic cancer cell line using flow cytometric analysis, supporting the potential therapeutic relevance of targeting NMDA receptors in these cells [12]. This finding provided a rationale for exploring the effects of DX on pancreatic cancer cell biology, particularly in relation to apoptosis and EMT.

Beyond its NMDA receptor antagonism, recent work indicates that dextromethorphan may also modulate the tumor microenvironment [13]. Notably, DXM inhibited trafficking of TGF-β–related proteins and reduced fibrillar collagen deposition in preclinical models, a mechanism potentially relevant to the dense desmoplastic stroma characteristic of pancreatic cancer. In parallel, the drug-repurposing literature has discussed DXM among sigma-1 receptor-targeting agents of oncologic interest, and contemporary reviews on NMDA receptor antagonists further underscore the role of glutamatergic signaling in cancer. Moreover, DXM combined with metformin synergistically attenuated nicotine-enhanced cancer-initiating properties in esophageal squamous cell carcinoma models, supporting combination approaches with repurposed agents [14,15,16].

The present study aimed to investigate the effects of DX, both alone and in combination with gemcitabine, on apoptosis and EMT-related mechanisms in the PANC-1 pancreatic cancer cell line. Through MTT cytotoxicity assays, immunocytochemistry, flow cytometry, and gene expression analyses, we sought to elucidate whether DX could enhance apoptosis and inhibit metastatic processes in vitro. This research could pave the way for further in vivo and clinical studies evaluating DX as a potential adjunct in pancreatic cancer therapy.

## 2. Results

### 2.1. Cell Viability Analysis

The cytotoxic effects of DX, GEM, and their combinations on PANC-1 cell viability were assessed using the MTT assay after 24, 48, and 72 h of treatment. Dose-dependent and time-dependent reductions in cell viability were observed for both agents.

Exposure to DX resulted in significant decreases in cell viability at concentrations ≥150 µM after 48 h and at all tested concentrations (25–200 µM) after 72 h (*p* < 0.001). At 72 h, viability dropped to 46.04% at 125 µM and to 33.62% at 200 µM. The IC_50_ value was determined as 105.6 µM at 72 h. These findings confirmed that DX exerts a strong, concentration-dependent cytotoxic effect on PANC-1 cells.

GEM exposure did not induce significant cytotoxicity at 24 h; however, cell viability significantly decreased at concentrations ≥250 µM after 48 h and at concentrations ≥1 µM after 72 h (*p* < 0.001). At 72 h, cell viability was 44.63% at 200 µM and 34.58% at 500 µM. The calculated IC_50_ value for GEM was 57.53 µM at 72 h.

Although 5-FU also decreased viability in a concentration-dependent manner, its IC_50_ value at 72 h (43.24 µM) was higher than GEM, and its effects were less consistent. Consequently, GEM was selected for further combination studies with DX.

The cytotoxic effect of DMSO, used as the solvent for DX, was assessed independently. DMSO concentrations corresponding to 50–400 µM DX (0.1–0.8%) induced minor but statistically significant reductions in viability only at the higher end. However, DX-treated groups showed significantly lower viability than DMSO controls, confirming that the cytotoxic effect was attributable to DX itself (*p* < 0.05–*p* < 0.001).

**Figure 1:**
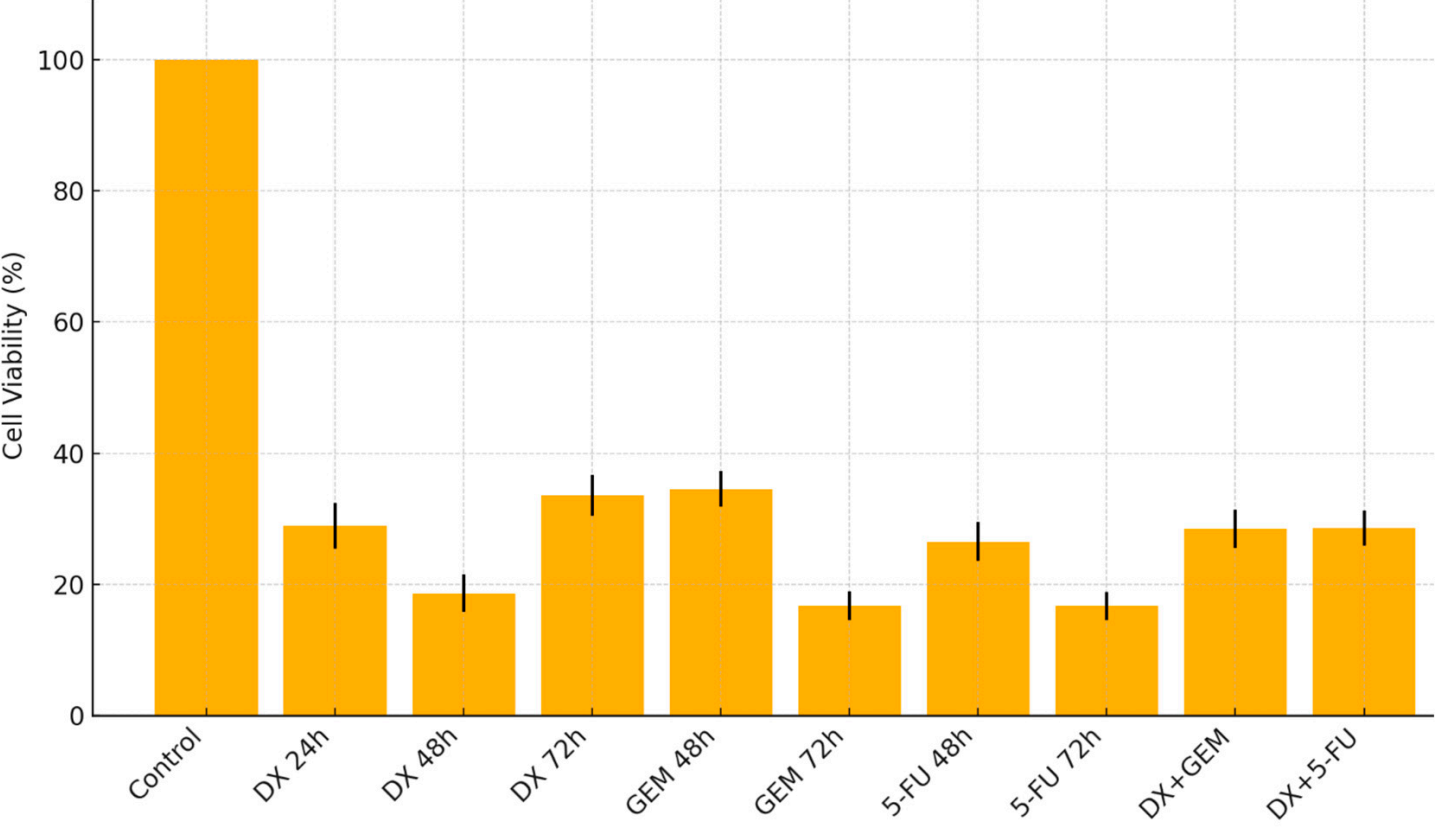
Effect of DX, GEM, 5-FU, and their combinations on PANC-1 cell viability (MTT Assay).

The combined treatment of DX and GEM (½ IC_50_ of each) showed a more pronounced reduction in cell viability compared to either drug alone. For example, 50 µM DX + 12.5 µM GEM reduced viability to 24.7% (*p* < 0.001 vs. control and single-agent groups). Tukey’s post hoc analysis confirmed significant differences between GEM-only and combination groups at corresponding concentrations (e.g., *p* = 0.015 for GEM 50 µM vs. GEM 50 µM + DX 200 µM) (Table 1, Figure 1).

IC_50_ values of active substances were shown in Table 2. The IC_50_ values for dextromethorphan showed a time-dependent decreasing trend. Accordingly, the IC_50_ was determined to be 280.4 µM at 24 h, 163.2 µM at 48 h, and 105.6 µM at 72 h of exposure. For gemcitabine, the IC_50_ value was calculated only after 72 h of incubation and was found to be 57.53 µM, indicating a strong cytotoxic effect in a relatively short period. As for 5-FU, the IC_50_ value was 340.1 µM at 48 h, which decreased to 43.24 µM after 72 h of incubation (Table 2).

To quantitatively assess drug interaction, we calculated the coefficient of drug interaction (CDI = AB/(A × B)) using 72 h viable-cell fractions. For DX 50 µM + GEM 12.5 µM, CDI was 1.003, consistent with an approximately additive effect under Bliss independence (observed 24.93% vs. expected 24.86%). Nevertheless, the combination produced a 15.4-point lower viability compared with the best single agent (HSA improvement), alongside the highest early/late apoptosis rates among groups (Table S1, Figure S1), corroborating the enhanced anti-tumor activity of the combination.

### 2.2. Flow Cytometry Analysis

**Figure 2:**
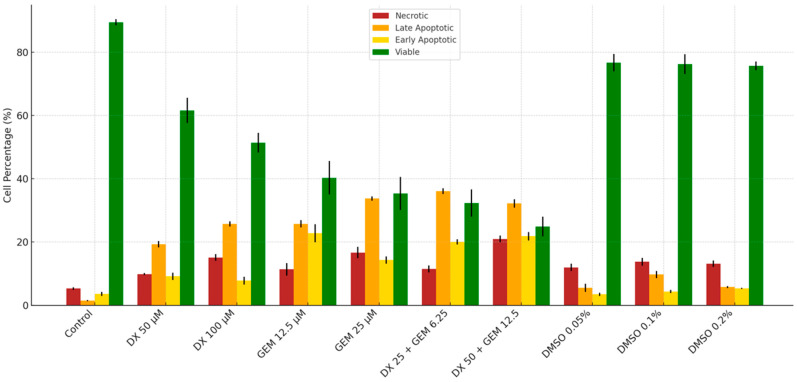
Apoptosis and viability profile in PANC-1 cells after 72 h (flow cytometry).

Flow Cytometry Analysis of apoptosis in PANC-1 cells was shown in Table 3. The percentage of viable cells was significantly reduced in all treatment groups compared to the control (*p* < 0.001), with the lowest viability observed in the DX 50 µM + GEM 12.5 µM combination group (24.93 ± 3.12%). In contrast, control cells exhibited the highest viability (89.53 ± 0.97%). Early apoptotic cell percentages were markedly increased in the DX and GEM groups individually, and even more prominently in the combination groups, reaching up to 21.83 ± 1.32% in the DX 50 µM + GEM 12.5 µM group. Late apoptotic cells followed a similar trend, with a peak at 36.10 ± 0.93% in the DX 25 µM + GEM 6.25 µM group. Additionally, necrotic cell percentages were significantly elevated in the combination groups compared to the control (*p* < 0.001), with the highest necrosis observed in the DX 50 µM + GEM 12.5 µM group (20.97 ± 1.04%) (Table 3, Figure 2).

### 2.3. Immunocytochemical (ICC) Analysis

**Figure 3:**
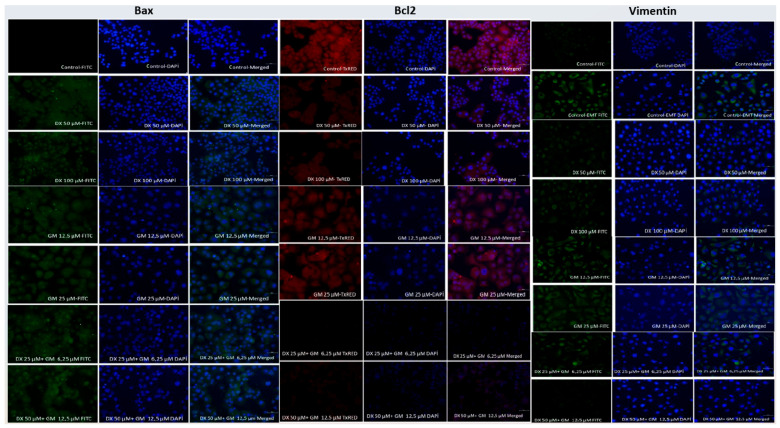
Immunocytochemical protein expression levels of Bax, Bcl-2, and Vimentin in PANC-1 cells after 72 h. Green fluorescence (FITC) indicates Bax and Vimentin protein expression, red fluorescence (TRITC) represents Bcl-2 protein expression, and blue fluorescence (DAPI) corresponds to nuclear counterstaining. Merged images show the overlay of protein-specific staining with nuclei, enabling visualization of expression patterns and localization within the cells. Scale bar = 100 µm.

ICC analysis of Bax, Bcl-2, and Vimentin expression in PANC-1 cells is shown in Table 4. A statistically significant increase in Bax-positive cells per unit area was observed in all treatment groups compared to the control (*p* < 0.001). Notably, the combination of DX 50 µM + GEM 12.5 µM yielded the highest Bax expression (62.0 cells/unit area), while the control group had the lowest (15.0 cells/unit area). Conversely, Bcl-2 and Vimentin expressions showed a significant reduction in treated groups. The Bcl-2-positive cell count decreased from 60.0 in the control to 19.0 in the DX 50 µM + GEM 12.5 µM group. Similarly, the Vimentin-positive cell count decreased significantly from 70.0 to 28.0 cells/unit area (Table 4, Figure 3).

**Figure 4:**
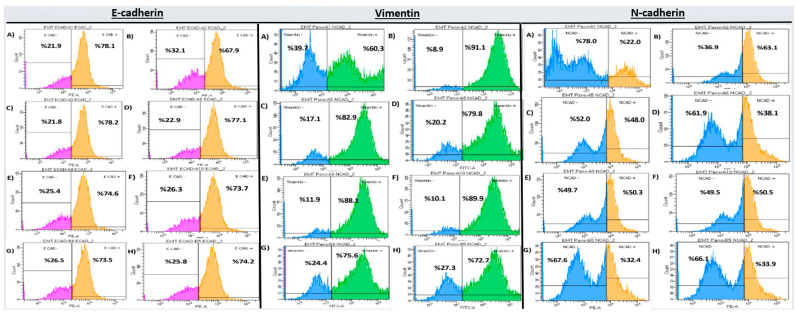
EMT marker expression in PANC-1 cells after 72 h treatment (flow cytometry). Subpanels show: E-cadherin (**A**–**H**), Vimentin (**A**–**H**), and N-cadherin (**A**–**H**). Flow cytometry histograms correspond to the quantitative data presented in Table 5, where groups sharing the same letter do not differ significantly, and groups marked with different letters show statistically significant differences (*p* < 0.05).

Flow Cytometry Analysis of EMT markers in PANC-1 cells is shown in Table 5. E-cadherin levels significantly increased in all treated groups compared to the EMT control (*p* < 0.001), with the highest expression observed in the DX 50 µM + GEM 12.5 µM group (83.84 ± 0.65), indicating a reversal of EMT. In contrast, mesenchymal markers Vimentin and N-cadherin were significantly reduced in all treatment groups (71.04 ± 1.17 and 30.47 ± 0.72, respectively) (Table 5, Figure 4).

### 2.4. qRT-PCR Gene Expression Analysis

qRT-PCR Gene Expression Analysis is shown in Table 6. Bax expression levels were significantly elevated in all treatment groups compared to the EMT control, with the highest expression in the DX 50 µM + GEM 12.5 µM group (2.1-fold), indicating enhanced pro-apoptotic activity (*p* < 0.05). Conversely, Bcl-2 and XIAP expression levels, which are anti-apoptotic genes, were significantly downregulated, particularly in the combination group (0.3- and 0.6-fold, respectively; *p* < 0.001 for XIAP). In terms of EMT-related gene expression, E-cadherin was upregulated in all treated groups, especially in the DX 50 µM + GEM 12.5 µM combination (2.0-fold), indicating a partial reversal of EMT. However, no statistically significant differences were observed among treatment groups for the mesenchymal markers N-cadherin, Vimentin, and Snail, as all groups shared the same superscript letter (*p* > 0.05) (Table 6).

## 3. Discussion

In this study, we investigated the effects of DX, both alone and in combination with GEM, on apoptosis and EMT in PANC-1 pancreatic cancer cells. Our findings demonstrated that DX exhibited intrinsic cytotoxic activity and, when combined with GEM, significantly enhanced apoptotic responses and inhibited EMT marker expression, suggesting a potential role as an adjuvant agent in pancreatic cancer therapy.

MTT assay results indicated a time- and dose-dependent cytotoxic effect of DX, with the 72 h IC_50_ value (105.6 µM) being markedly lower than that observed at 24 h (280.4 µM). This result was consistent with increasing cytotoxic potency upon prolonged exposure. GEM also showed potent time-dependent cytotoxicity, which was in agreement with previous studies reporting its efficacy in inducing apoptosis and cell cycle arrest in pancreatic cancer cells [17,18,19,20]. Samanta et al. demonstrated that gemcitabine exposure over 72 h significantly reduced pancreatic tumor cell viability in vitro, particularly when combined with other agents [21]. Furthermore, Kim et al. confirmed that prolonged gemcitabine exposure increased apoptotic indices and caspase activation in PANC-1 and MIA PaCa-2 cells, highlighting the relevance of treatment duration to cytotoxic efficacy [22].

The combination of DX and GEM at sub-IC_50_ concentrations led to a significantly greater reduction in cell viability compared to either agent alone. Dalisay et al. demonstrated that combining NMDA receptor antagonists with chemotherapy enhanced cytotoxic effects in glioblastoma models [23]. Our results suggested that DX may act as a sensitizer to GEM, potentially through modulation of apoptotic and survival signaling pathways. Similar synergistic outcomes have been reported by Zhao et al., who showed that inhibition of glutamate signaling sensitized colorectal cancer cells to 5-FU via enhanced apoptosis [24]. Likewise, Du et al. found that pharmacologic blockade of NMDA receptors reduced proliferation and increased the cytotoxicity of anticancer agents in neuroblastoma and lung carcinoma cells [25], supporting the hypothesis that DX may potentiate GEM’s efficacy by disrupting glutamate-mediated pro-survival mechanisms.

Flow Cytometry Analysis confirmed that both early and late apoptotic cell populations were significantly increased following treatment, particularly in the DX 50 µM + GEM 12.5 µM group. Viable cell percentages were concurrently reduced. These observations were consistent with the study by Chen et al., who identified functional NMDA receptor expression in PANC-1 cells and linked their modulation to cell survival [7]. Zhao et al. showed that NMDA receptor blockade induced apoptosis in hepatocellular carcinoma cells through mitochondrial depolarization and caspase activation [24]. Gallo et al. demonstrated that suppression of NMDA receptor signaling in breast cancer cells led to a significant increase in annexin V-positive apoptotic populations, supporting the notion that targeting this pathway promotes programmed cell death [9]. These findings reinforce the hypothesis that dextromethorphan may sensitize cancer cells to chemotherapeutic-induced apoptosis by interfering with NMDA receptor-mediated survival signaling.

Immunocytochemical staining further supported the induction of apoptosis. The number of Bax-positive cells per unit area increased significantly in all treatment groups, with the combination group showing the highest level. In contrast, the anti-apoptotic marker Bcl-2 and the mesenchymal marker Vimentin were significantly decreased. Koda et al. reported that inhibition of NMDA receptor signaling increased pro-apoptotic markers and reduced anti-apoptotic ones in colon cancer cells [26]. Likewise, Maniam et al. demonstrated that combined chemotherapeutic treatments enhanced Bax expression while reducing Bcl-2 in pancreatic tumor models, thereby shifting the Bax/Bcl-2 ratio toward apoptosis [27]. Zhang et al. observed that inhibition of EMT markers such as Vimentin coincided with increased apoptosis and decreased metastatic potential in breast cancer cells treated with glutamate receptor modulators [28]. These findings further support our results, indicating that the combined treatment strategy targets both apoptotic and mesenchymal pathways to effectively reduce tumor cell viability.

EMT marker analysis by flow cytometry revealed increased E-cadherin and decreased Vimentin and N-cadherin expression levels in the treatment groups. The most significant modulation was observed in the combination group, suggesting that co-administration of DX and GEM effectively reversed EMT. Chuang et al. demonstrated that EMT inhibition reduced metastatic potential in pancreatic cancer models [29].

Although qRT-PCR analysis showed upregulation of Bax and E-cadherin and downregulation of Bcl-2 and XIAP, most mesenchymal marker gene expression levels (N-cadherin, Vimentin, Snail) did not differ significantly between groups. The discrepancy between protein and mRNA levels may have resulted from post-transcriptional modifications or delayed gene expression responses. Importantly, XIAP expression was significantly reduced in the combination group, supporting the activation of the intrinsic apoptotic pathway [30,31].

Our findings align with emerging evidence that DXM may exert anticancer effects through multiple axes, including glutamatergic pathway interference and microenvironmental remodeling. The observation that DXM reduces collagen deposition via TGF-β–related trafficking inhibition suggests potential relevance in PDAC desmoplasia, while repurposing analyses of sigma-1 receptor ligands and broader NMDA antagonist reviews place DXM within a mechanistic framework for oncology. Prior studies also indicated synergy of DXM with metformin in nicotine-driven tumor models, consistent with our combination-therapy rationale [13,14,15,16]. Moreover, recent advances in nanotechnology and functional biomaterials provide promising directions for enhancing the therapeutic potential of anticancer strategies. For instance, nanocarrier-based systems have been shown to improve drug delivery, tumor penetration, and to overcome microenvironmental barriers [32]. Integrating such approaches with repurposed drugs like DXM could further amplify therapeutic efficacy in pancreatic cancer.

Compared to previous studies, this investigation provides several novel insights. While earlier research has established the individual anticancer effects of gemcitabine [33] and has explored the role of NMDA receptors in various cancer types [34,35,36,37], few studies have evaluated the repurposing of dextromethorphan (DX) as an adjuvant in pancreatic cancer treatment [38,39]. Notably, this is the first study to demonstrate that DX, when combined with gemcitabine, synergistically induces apoptosis and suppresses EMT in PANC-1 cells. Unlike the study by Dalisay et al., which focused on glioblastoma models, our findings extend the relevance of NMDA receptor modulation to pancreatic cancer [23]. Furthermore, although North et al. confirmed NMDA receptor expression in PANC-1 cells, they did not explore its therapeutic targeting [12]. Our study builds on this by functionally validating the apoptotic and anti-EMT effects of NMDA receptor blockade. In contrast to other EMT-focused studies, such as that by Zhang et al., which investigated transcription factor inhibition, we targeted membrane receptor signaling with a clinically available antitussive drug, offering a more translatable approach [28]. This combination of mechanistic insight and therapeutic potential marks a key advancement over the previously published literature.

### Limitations of the Study

Despite the promising results, this study has some limitations. First, all experiments were conducted in vitro using a single pancreatic cancer cell line (PANC-1). While these findings provide valuable mechanistic insights, they may not fully represent the complex tumor microenvironment or heterogeneity seen in clinical pancreatic cancer. Second, although we observed significant changes in protein expression levels of EMT and apoptotic markers, qRT-PCR analysis revealed limited changes at the mRNA level for some genes, suggesting that post-transcriptional regulation may be involved. This discrepancy warrants further investigation using techniques such as Western blotting or transcriptome-wide profiling. In addition, although apoptosis was confirmed by Annexin V/7-AAD flow cytometry, immunocytochemistry, and qRT-PCR analyses, Western blot validation of cleaved caspase-3 or cleaved PARP was not performed in this study. Future investigations will incorporate these analyses to provide further mechanistic confirmation of the apoptotic pathway. Moreover, functional validation using a pan-caspase inhibitor such as Z-VAD-FMK was not performed. Future studies will incorporate such experiments to further verify that the observed cytotoxicity is predominantly caspase-dependent apoptosis. Future studies will incorporate such experiments to further verify that the observed cytotoxicity is predominantly caspase-dependent apoptosis. Furthermore, although EMT modulation was demonstrated by both protein (flow cytometry, immunocytochemistry) and gene expression analyses, functional migration or invasion assays were not performed. Future studies incorporating wound healing or transwell assays will be necessary to confirm the functional impact of DX on EMT. Third, the molecular mechanism underlying the synergy between dextromethorphan and gemcitabine was not elucidated in detail. While the observed effects suggest NMDA receptor involvement, receptor-specific assays or pharmacological antagonism/agonism experiments were not performed. Finally, the lack of in vivo validation is a major limitation. Animal studies are necessary to evaluate the bioavailability, pharmacokinetics, and therapeutic efficacy of this drug combination in a physiological context. Future studies incorporating multiple cell lines, in vivo models, and mechanistic pathway analysis will be essential to validate and expand upon these findings.

## 4. Materials and Methods

### 4.1. Study Design and Cell Culture

This in vitro experimental study was conducted at the Erciyes University Genome and Stem Cell Center, supported by the Erciyes University Scientific Research Projects Unit (Project Code: TDK-2018-8652). The human pancreatic cancer cell line PANC-1 (ATCC^®^ CRL-1469™) was used throughout the experiments. Cells were cultured in high-glucose Dulbecco’s Modified Eagle’s Medium (DMEM-HG) supplemented with 10% fetal bovine serum (FBS), 1% penicillin–streptomycin, and 1% L-glutamine at 37 °C in a humidified incubator with 5% CO_2_.

### 4.2. Drug Preparation

Dextromethorphan hydrobromide hydrate (BioVision), gemcitabine hydrochloride (ADOOQ), and 5-fluorouracil (5-FU) were used. Stock solutions were prepared as follows:

- **Dextromethorphan**: 42.5 mM in DMSO;
- **Gemcitabine**: 20 mM in DPBS;
- **5-FU**: diluted to 2.5 mM in culture medium from a 384 mM stock.

All drug solutions were filtered through 0.2 µm sterile filters before use.

### 4.3. Cytotoxicity Assay (MTT)

To determine the IC_50_ values of each drug, MTT assay was conducted in 96-well plates. PANC-1 cells were seeded at 6 × 10^3^ cells/well and incubated overnight. After cell attachment, drugs were applied in increasing concentrations for 24, 48, and 72 h. MTT solution (10 µL of 5 mg/mL) was added, and plates were incubated for 3 h. Formazan crystals were dissolved in 100 µL DMSO, and absorbance was measured at 560 nm (reference: 750 nm) using a plate reader (Promega Glomax, GloMax^®^, Promega Corporation, Madison, WI, USA). IC_50_ values were calculated using GraphPad Prism 8 software. Coefficient of Drug Interaction (CDI) was calculated as AB/(A × B) to evaluate the nature of the combination effects, where AB is the observed viability of the combination group, and A and B represent the relative viabilities of each single agent.

### 4.4. Experimental Groups

Based on the MTT assay results, experimental groups were determined using the IC_50_ values and their fractions. The IC_50_ values at 72 h were 105.6 µM for DX and 57.53 µM for GEM. To evaluate potential synergistic or additive effects without inducing maximal cytotoxicity from either drug alone, we selected sub-IC_50_ concentrations corresponding to ½, ¼, and ⅛ of the IC_50_ values. For example, DX 50 µM (~½ IC_50_) was combined with GEM 12.5 µM (~¼ IC_50_). This strategy enabled the assessment of combination effects at clinically relevant, non-maximal cytotoxic doses and ensured that the observed reductions in viability and changes in apoptosis/EMT markers could be attributed to the interaction between DX and GEM rather than excessive toxicity from a single agent. Accordingly, the experimental groups included the following:

- **DX alone** (IC_50_ and ½ IC_50_);
- **Gemcitabine alone** (IC_50_ and ½ IC_50_);
- **DX + gemcitabine combination** (½, ¼, and ⅛ of IC_50_ values);
- **Control and TGF-β-induced EMT groups**.

### 4.5. EMT Induction

EMT was induced by treating PANC-1 cells with transforming growth factor-beta 1 (TGF-β1) in media containing varying concentrations of FBS. Optimal EMT conditions were determined by assessing cellular morphology under an inverted microscope, selecting 5 ng/mL TGF-β1 with 10% FBS as the standard.

### 4.6. Flow Cytometry for Apoptosis

Apoptosis was evaluated using Annexin V-FITC/7-AAD staining. Cells were treated with drugs for 72 h, harvested by trypsinization, and washed. Cells were stained with 5 µL Annexin V-FITC and 5 µL 7-AAD, incubated for 15 min, and diluted with 400 µL Cell Wash Buffer. Analysis was performed using a BD FACS Aria III cytometer with BD FACSDiva™ Software v 8.0.

### 4.7. Flow Cytometry for EMT Markers

Expression of EMT-related markers E-cadherin, N-cadherin, and vimentin was assessed by flow cytometry. Cells were stained with fluorochrome-conjugated antibodies:

- **Vimentin-FITC** (1:250);
- **N-cadherin-PE** (1:200);
- **E-cadherin-PE** (1:250).

Data were acquired using BD FACS Aria III.

### 4.8. Immunocytochemistry (ICC)

Cells were seeded in 24-well plates with glass coverslips and treated for 72 h. After washing with DPBS, cells were fixed with 3.7% paraformaldehyde and permeabilized with 0.25% Triton X-100 for Bax and Bcl-2 staining. Blockage was performed using 1% BSA and 22.5 mg/mL glycine in PBST. Primary antibodies used include the following:

- **Bax (1:200, Bioss Inc., Woburn, MA, USA)**;
- **Bcl-2 (1:50, Thermo Fisher Scientific Inc., Waltham, MA, USA)**.

Secondary antibodies:

- **Donkey anti-Rb 488 for Bax (1:500)**;
- **Goat anti-Rb 594 for Bcl-2 (1:500)**.

Fluorochrome-conjugated antibodies were used for the following:

- **Vimentin-FITC, N-cadherin-PE, and E-cadherin-PE**.

After staining, cells were mounted with DAPI-containing mounting medium and imaged using a Nikon Eclipse Ti fluorescence microscope (Nikon Instruments Inc., Melville, NY, USA).

### 4.9. Gene Expression Analysis (qRT-PCR)

Total RNA was extracted using the NucleoSpin RNA kit and quantified by Nanodrop. cDNA synthesis was performed using the OneScript Plus cDNA Synthesis Kit (ABM, Richmond, BC, Canada). Gene expression levels of Bax, Bcl-2, XIAP, E-cadherin, N-cadherin, Vimentin, and Snail were analyzed using SYBR Green qPCR (Roche LightCycler 480, Roche Diagnostics GmbH, Mannheim, Germany). GAPDH was used as the housekeeping gene. Expression data were analyzed using the ΔΔCt method.

### 4.10. Statistical Analysis

All statistical analyses were conducted using GraphPad Prism version 8.0 (GraphPad Software Inc., San Diego, CA, USA). Data were expressed as mean ± standard deviation (SD) from at least three independent experiments performed in triplicate. To compare the differences among multiple groups, One-Way Analysis of Variance (ANOVA) was applied, followed by Dunnett’s post hoc test to assess differences between treatment groups and the corresponding control (control or EMT control). In specific comparisons, such as between drug-treated and solvent control groups or in IC_50_ calculations, Student’s *t*-test was employed. A *p*-value < 0.05 was considered statistically significant.

## 5. Conclusions

In conclusion, this study provided evidence that DX, particularly in combination with GEM, promoted apoptosis and suppressed EMT-associated marker expression in PANC-1 pancreatic cancer cells. These dual effects addressed key mechanisms involved in pancreatic cancer progression: resistance to apoptosis and increased metastatic potential. Our findings supported the therapeutic potential of repurposing DX as a chemosensitizer and EMT modulator in pancreatic cancer.

## Figures and Tables

**Table 1:** Cell viability and cytotoxic concentrations in the MTT assay. n.s.: Not Significant.

Treatment	Viability (Mean ± SD)	Most Cytotoxic Conc. (µM)	Statistical Significance
DX (24 h)	28.99 ± 3.5	400	*p* < 0.001
DX (48 h)	18.67 ± 2.8	500	*p* < 0.001
DX (72 h)	33.62 ± 3.1	200	*p* < 0.001
GEM (24 h)	—	—	n.s.
GEM (48 h)	34.58 ± 2.7	500	*p* < 0.001
GEM (72 h)	16.72 ± 2.2	425	*p* < 0.001
5-FU (48 h)	26.57 ± 3.0	375	*p* < 0.001
5-FU (72 h)	16.72 ± 2.1	425	*p* < 0.001
DX + GEM (72 h)	28.5 ± 2.9	DX 50 + GEM 12.5	*p* < 0.001
DX + 5-FU (72 h)	28.6 ± 2.7	DX 50 + 5-FU 12.5	*p* < 0.001

**Table 2:** IC_50_ values of active substances (IC50: half-maximum inhibitory concentration 5-FU: 5-fluorouracil).

Agent	IC_50_ (24 h, µM)	IC_50_ (48 h, µM)	IC_50_ (72 h, µM)
Dextromethorphan	280.4	163.2	105.6
Gemcitabine	–	–	57.53
5-FU	–	340.1	43.24

**Table 3:** Flow cytometry analysis of apoptosis in PANC-1 cells (72 h treatment).

Group	Necrotic Cells	Late Apoptotic Cells	Early Apoptotic Cells	Viable Cells
	Mean ± SD			
Control	5.33 ± 0.40 ^a^	1.50 ± 0.17 ^a^	3.60 ± 0.61 ^a^	89.53 ± 0.97 ^a^
DX 50 µM	9.87 ± 0.32 ^b^	19.32 ± 0.99 ^b^	9.20 ± 1.15 ^b^	61.63 ± 4.00 ^b^
DX 100 µM	15.10 ± 1.04 ^c^	25.73 ± 0.85 ^b^	7.80 ± 1.25 ^b^	51.40 ± 3.10 ^b^
GEM 12.5 µM	11.37 ± 1.94 ^bc^	25.75 ± 1.13 ^b^	22.77 ± 2.84 ^c^	40.33 ± 5.26 ^c^
GEM 25 µM	16.67 ± 1.80 ^c^	33.75 ± 0.66 ^c^	14.30 ± 1.14 ^b^	35.33 ± 5.22 ^c^
DX 25 + GEM 6.25 µM	11.50 ± 1.10 ^b^	36.10 ± 0.93 ^c^	20.03 ± 0.81 ^c^	32.33 ± 4.32 ^c^
DX 50 + GEM 12.5 µM	20.97 ± 1.04 ^d^	32.20 ± 1.33 ^c^	21.83 ± 1.32 ^c^	24.93 ± 3.12 ^d^
DMSO %0.05	12.00 ± 1.15 ^b^	5.57 ± 1.29 ^a^	3.50 ± 0.50 ^a^	76.73 ± 2.78 ^a^
DMSO %0.1	13.77 ± 1.26 ^b^	9.74 ± 1.15 ^a^	4.37 ± 0.53 ^a^	76.32 ± 3.12 ^a^
DMSO %0.2	13.17 ± 1.02 ^b^	5.77 ± 0.31 ^a^	5.37 ± 0.21 ^a^	75.70 ± 1.39 ^a^
*p*-value (vs. Control)	<0.001	<0.001	<0.001	<0.001

Groups sharing the same superscript letter do not differ significantly. Groups marked with different letters show statistically significant differences (*p* < 0.05).

**Table 4:** ICC analysis of Bax, Bcl-2, and Vimentin expression in PANC-1 cells (72 h).

Group	Bax	Bcl-2	Vimentin
	Cells/Unit Area		
Control	15.0 ^a^	60.0 ^a^	70.0 ^a^
DX 50 µM	42.0 ^b^	32.0 ^b^	45.0 ^b^
DX 100 µM	51.0 ^c^	28.0 ^b^	41.0 ^b^
GEM 12.5 µM	47.0 ^c^	29.0 ^b^	50.0 ^b^
GEM 25 µM	52.0 ^c^	25.0 ^b^	48.0 ^b^
DX 25 + GEM 6.25 µM	57.0 ^d^	21.0 ^c^	34.0 ^c^
DX 50 + GEM 12.5 µM	62.0 ^e^	19.0 ^c^	28.0 ^c^
*p*-value (vs. Control)	<0.001	<0.001	<0.001

Groups sharing the same superscript letter do not differ significantly. Groups marked with different letters show statistically significant differences (*p* < 0.05).

**Table 5:** Flow Cytometry Analysis of EMT markers in PANC-1 cells (72 h).

Group	E-Cadherin	Vimentin	N-Cadherin
	Mean ± SD		
Control EMT	68.97 ± 0.97 ^a^	91.00 ± 1.85 ^a^	62.47 ± 0.93 ^a^
DX 50 µM	78.27 ± 1.01 ^b^	83.93 ± 2.03 ^b^	48.71 ± 1.02 ^b^
DX 100 µM	78.61 ± 1.02 ^b^	78.95 ± 2.94 ^b^	45.36 ± 1.08 ^b^
GEM 12.5 µM	76.15 ± 1.67 ^b^	80.37 ± 3.01 ^a^	42.90 ± 1.04 ^b^
GEM 25 µM	76.17 ± 1.35 ^b^	80.71 ± 2.93 ^a^	40.77 ± 0.94 ^b^
DX 25 + GEM 6.25 µM	80.43 ± 0.74 ^b^	73.44 ± 1.03 ^b^	32.07 ± 0.73 ^b^
DX 50 + GEM 12.5 µM	83.84 ± 0.65 ^b^	71.04 ± 1.17 ^b^	30.47 ± 0.72 ^b^
*p*-value (vs. EMT control)	<0.001	<0.001	<0.001

Groups sharing the same letter do not differ significantly. Groups marked with different letters show statistically significant differences (*p* < 0.05).

**Table 6:** qRT-PCR Gene Expression Analysis (fold change relative to EMT control).

Group	Bax	Bcl-2	XIAP	E-Cadherin	N-Cadherin	Vimentin	Snail
EMT control	1.0 ^a^	1.0 ^a^	1.00 ^a^	1.0 ^a^	1.00 ^a^	1.00 ^a^	1.00 ^a^
DX 50 µM	1.2 ^a^	0.8 ^a^	0.95 ^a^	1.4 ^a^	0.85 ^a^	0.90 ^a^	0.95 ^a^
DX 100 µM	1.5 ^a^	0.7 ^a^	0.90 ^a^	1.5 ^a^	0.80 ^a^	0.85 ^a^	0.90 ^a^
GEM 12.5 µM	1.6 ^a^	0.6 ^a^	0.85 ^a^	1.6 ^a^	0.75 ^a^	0.80 ^a^	0.85 ^a^
GEM 25 µM	1.7 ^a^	0.5 ^a^	0.75 ^a^	1.7 ^a^	0.70 ^a^	0.75 ^a^	0.80 ^a^
DX 25 + GEM 6.25 µM	1.9 ^a^	0.4 ^a^	0.65 ^a^	1.9 ^a^	0.65 ^a^	0.70 ^a^	0.75 ^a^
DX 50 + GEM 12.5 µM	2.1 ^a^	0.3 ^a^	0.60 ^b^	2.0 ^a^	0.60 ^a^	0.65 ^a^	0.70 ^a^
*p*-value (DX + GEM vs. EMT control)	>0.05	>0.05	<0.001	>0.05	>0.05	>0.05	>0.05

Groups sharing the same letter do not differ significantly. Groups marked with different letters show statistically significant differences (*p* < 0.05).

## Data Availability

Data is available upon request to the corresponding author.

## Supplementary Materials

The following supporting information can be downloaded at: https://www.mdpi.com/article/10.3390/ijms26178151/s1.
