# Supplementary material for: Dextromethorphan Enhances Apoptosis and Suppresses EMT in PANC-1 Pancreatic Cancer Cells: Synergistic Effects with Gemcitabine
Source: Int J Mol Sci. 2025 Aug 22;26(17):8151. doi: 10.3390/ijms26178151 (PMC12428249; doi:10.3390/ijms26178151)
Supplement: Supplementary file 1 [file ijms-26-08151-s001.zip › ijms-3812801-supplementary.pdf]

Table S1. Coefficient of Drug Interaction (CDI) and related synergy metrics for DX + GEM (72 h).

Treatment Group	Viability (%)	Expected (A × B, %)	CDI	HSA Improvement (%)	Interpretation
DX 50 μM	61.63	–	–	–	Single agent
GEM 12.5 μM	40.33	–	–	–	Single agent
DX 50 + GEM 12.5 μM	24.93	24.86	1.003	15.40	Additive by CDI, improved over best single agent

Comparison of observed viability (24.93%) for the combination of DX 50 μM + GEM 12.5 μM with the expected viability under Bliss independence (24.86%) at 72 h. The close overlap indicates an additive effect (CDI = 1.003). Despite this, the combination reduced cell viability by 15.4 percentage points compared to the best single agent (HSA improvement), demonstrating enhanced efficacy over monotherapies (Figure S1).

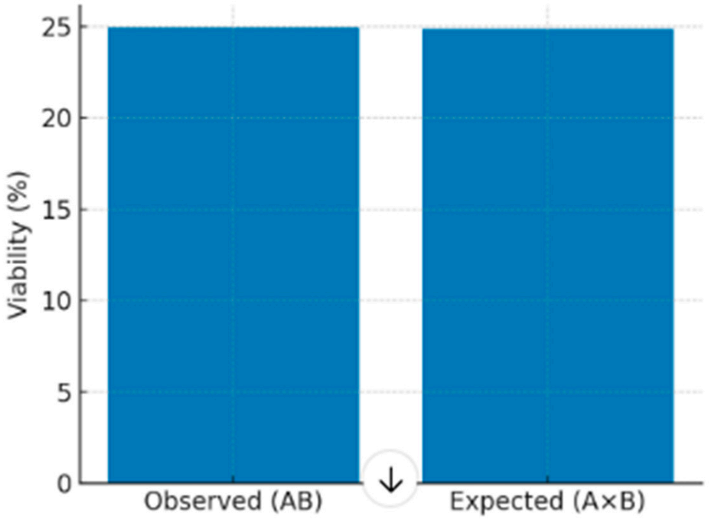

Figure S1. Observed vs. Expected Cell Viability for DX + GEM (72 h).
